# A Novel Framework Using Deep Auto-Encoders Based Linear Model for Data Classification

**DOI:** 10.3390/s20216378

**Published:** 2020-11-09

**Authors:** Ahmad M. Karim, Hilal Kaya, Mehmet Serdar Güzel, Mehmet R. Tolun, Fatih V. Çelebi, Alok Mishra

**Affiliations:** 1 Computer Engineering Department, AYBU, Ankara 06830, Turkey; ahmad.mozaffer.karim@gmail.com (A.M.K.); hilalkaya@ybu.edu.tr (H.K.); fatihvcelebi@gmail.com (F.V.Ç.); 2 Computer Engineering Department, Ankara University, Ankara 06830, Turkey; mguzel@ankara.edu.tr; 3 Computer Engineering Department, Konya Food and Agriculture University, Konya 42080, Turkey; mehmet.tolum@gidatarim.edu.tr; 4 Faculty of Logistics, Molde University College-Specialized University in Logistics, 6402 Molde, Norway; 5 Software Engineering Department, Atilim University, Ankara 06830, Turkey

**Keywords:** deep sparse auto-encoders, medical diagnosis, linear model, data classification, PSO algorithm

## Abstract

This paper proposes a novel data classification framework, combining sparse auto-encoders (SAEs) and a post-processing system consisting of a linear system model relying on Particle Swarm Optimization (PSO) algorithm. All the sensitive and high-level features are extracted by using the first auto-encoder which is wired to the second auto-encoder, followed by a Softmax function layer to classify the extracted features obtained from the second layer. The two auto-encoders and the Softmax classifier are stacked in order to be trained in a supervised approach using the well-known backpropagation algorithm to enhance the performance of the neural network. Afterwards, the linear model transforms the calculated output of the deep stacked sparse auto-encoder to a value close to the anticipated output. This simple transformation increases the overall data classification performance of the stacked sparse auto-encoder architecture. The PSO algorithm allows the estimation of the parameters of the linear model in a metaheuristic policy. The proposed framework is validated by using three public datasets, which present promising results when compared with the current literature. Furthermore, the framework can be applied to any data classification problem by considering minor updates such as altering some parameters including input features, hidden neurons and output classes.

## 1. Introduction

Deep learning (DL) is a new paradigm of neural networks, which is employed in different fields such as image classification and recognition, medical imaging and robotics etc. The deep auto-encoder (DAE) is also a popular deep learning technique and has been recently adapted to various applications in different fields [1,2,3,4]. Bhatkoti and Paul propose a new framework for Alzheimer’s disease diagnosis based on deep learning and the KSA algorithm. In this application, the results of the modified approach are compared to the non-modified k-sparse method. The σKSA algorithm optimizes the competence of diagnosis compared to the previous research [5,6]. Tong et al. present a software defect prediction application by using the advantages of stacked denoising auto-encoders (SDAEs) and a two-stage ensemble (TSE). In the first step, SDAEs are used to learn the deep representations from the imitative software metrics. Moreover, a new ensemble learning method, TSE, is proposed to predict the label imbalance problem. The proposed method is trained and tested by using 12 NASA benchmark test data to show the effectiveness of the SDAEsTSE system, which is significantly effective for software defect prediction [7].

Kuo et al. (2017) propose a stacked denoising auto-encoder for building a deep network for student dropout prediction. The system is trained with recent years’ data and is used to estimate the results of the current year for counseling in order to warn of students who might drop out [8]. 

Another leading study trains an auto-encoder neural network to encode and decode a geochemical data with unidentified composite multivariate possibility distributions. During the training, rare event examples contribute to the deep auto-encoder network. These examples can be classified by the trained network as abnormal examples due to their reasonably greater reconstructed mistakes. The Southwestern Fujian district in China is selected as a case research field [8]. 

Han et al. present some ideas of a deep sparse auto-encoder mixed with compressed sensing (CS) theory, which can enhance the compacted selection process of CS with compressing of the sparse auto-encoder in deep learning. The innovative CS theory does not provide any function of autonomic instruction, so they present the notion of a stacked auto-encoder of a deep neural network to optimize the theory. At that point, they compute the mistakes between the retrieval of the input and output features. By adjudicating the achieved error and the suitable error, the stacked auto-encoder compressed sensing model can select separately the best suitable sparsity and the best suitable length of dimension vector [9]. Salaken et al. propose a deep auto-encoder classification technique which primarily learns high-level features and then trains an artificial neural network (ANN) by employing these learned features. Experimental results prove that the technique offers satisfactory results when compared with other state-of-the arts classifiers when trained with the same features and the training set [10]. Khatab et al. present a novel technique which takes advantage of deep learning and deep extracted features by employing an auto-encoder to enhance the localization achievement in the feature learning and the classification. Moreover, the fingerprint dataset also needs to be reorganized, so the authors increase the training data number, so as to enhance the localization achievement, progressively. Experiments show that the presented technique supplies an important enhancement in localization achievement by using deep features extracted by an auto-encoder and increasing the training data number [11]. 

In addition to the deep auto-encoder neural network, a convolutional neural network has effective applications. Khan et al. offer a new convolutional neural network and random forest estimator to categorize the complex time series input, identifying whether it agrees with a breathing activity. Furthermore, the authors collect a comprehensive dataset for training the proposed method and evolve reference benchmarks for future studies comprising the field. According to the obtained results, they conclude that convolutional neural networks mixed with passive radars show high potential for a taxonomy of human actions [12]. Tang et al. propose a novel method, involving a preprocessing step, supported with two deep auto-encoders. Within the pre-processing stage, the input data are divided into segments, and then formal information is extracted so as to feed auto-encoders. It is claimed that this method produces acceptable results when compared with CNN-based feature learning approaches [13]. Yin et al. propose a new approach to explore an intrusion recognition system depending on a deep neural network. They propose a model for “intrusion recognition” based on recurrent neural networks (“RNN-IDS”). Furthermore, the system can achieve classification process as both a binary and multiclass classifier. The proposed model is compared with random forest, J48, SVM, ANN, and other machine learning techniques presented in earlier studies on the commonly used dataset. The experiments prove that “RNN-IDS” is actually appropriate for demonstrating a reliable classification model and outperforms well-known machine learning classification methods in binary and multiclass classification problems [14]. Yu et al. propose a technique to automatically classify the fetal facial standard plane (FFSP) by using a deep convolutional neural network (DCNN) method. The technique involves “16” convolutional layers, having small size “3 × 3” kernels, and also fully-connected layers (FCLs) layers. To reduce DCNN parameters, a “global average pooling” is adopted into the last pooling layer, which relieves the overfitting status and mends the achievement under fixed training data. The transfer learning technique followed by a data increase method, appropriate for FFSP, are executed to increase the classification accuracy gradually. Comprehensive experiments validate the benefit of the proposed approach through classical methods and the performance of DCNN to classify fetal facial standard plane for clinical detection [15]. 

Visual surveying of the large size of data has drawbacks and weaknesses. Visual investigation is time-consuming and may encounter conflicts in recognition, classification and detection processes, which are fundamental problems of large size of data. Therefore, many computer-aided diagnosis systems are proposed for data classification and processing by using machine learning techniques etc. Despite the researchers’ recent interest, it is still an open field and needs further solutions. This essentially motivates authors to contribute in this field. Accordingly, as aforementioned, this study introduces a general framework for data classification and processing issues. This framework is verified by employing a number of benchmark datasets in different fields. Overall, the main advantage of this framework is its remarkable experimental results when compared with former studies. Furthermore, the proposed framework can be used in any field with minimum effort by setting the model parameters based on the characteristics of the problem. 

Generally, the two sparse auto-encoders are utilized to diminish the dimension of input features and learn refined features. Those features are then classified by employing a Softmax layer. The whole model is stacked to provide a supervised training methodology. Then, the critical contribution is achieved by integrating a linear model, utilizing a metaheuristic algorithm for optimization, and is applied to enhance the deep sparse auto-encoder performance.

## 2. Literature Review 

Several studies related to three different datasets (epileptic seizure detection, cardiac arrhythmia and SPECTF classification) are analyzed and presented in Tables 10–12. Epileptic seizure is one of the most studied diseases in the field of computer-aided detection systems. Srinivasan et al. propose a new system based on time–frequency domain for feature extraction, and RNN were used to classify the features. The proposed method presents 99.60% accuracy as can be seen in [16]. Subasi and Ercelebi propose artificial neural network (ANN)-based wavelet transform (WT) and produce only 92% performance [17]. Subasi proposes a discrete WT based on a mixture of expert model, which presents only 94.5% performance [18]. Kannathal et al. propose a dynamic neuro-fuzzy inference system (ANFIS) based on entropy measures and produce 95% performance as can be seen in [19]. Tzallas et al. propose a new method based on time–frequency analysis and ANN which produces a high accuracy of 100% [20]. Polat and Güneş propose a fast Fourier transformation and decision tree (DT) which presents 98.72% performance [21]. Acharya et al. employ wavelet packet decomposition (WPD) to decompose segments and principal component analysis (PCA) to extract eigenvalues from the coefficients. Then, a supervised technique, namely, Gaussian mixture model (GMM) classifier, is employed to categorize the extracted features and obtain 99% accuracy [22]. Acharya et al. propose a combination of entropies, “HOS”, “Higuchi FD”, “Hurst exponent” and FC, and the proposed method offers “99.70%” accuracy [23]. Peker et al. propose a complex-value artificial neural network (CVANN) based on dual tree complex wavelet transformation (DTCWT). The proposed method presents 100% performance [24]. Karim et al. propose a new framework involving deep sparse auto-encoders (DSAE) utilizing the Taguchi optimization method, and the proposed method presents 100% accuracy [25]. Recently, Karim et al. modified the same framework by incorporating energy spectral density function, used to extract features, into a similar DSAE architecture. The results reveal that it outperforms many existing systems, especially in medical datasets [26].

Additionally, an important study in arrhythmias relying on spontaneous methods was recently offered, in which a model for estimation of cardiac arrhythmias is proposed [27]. The presented method applies two conventional supervised techniques (k-NN and SVM), respectively. The proposed method is validated and tested by employing the “UCI” dataset. While k-NN presents “73.8%” accuracy rate, SVM surprisingly achieves a 68.8% accuracy rate. Mustaqeem et al. propose a novel system for the recognition of arrhythmia, according to which, a wrapper algorithm is initially used to select effective features from the UCI dataset. Then, different classifiers, namely MLP, KNN, SVM, RFT and NB are combined with the proposed feature-extracted algorithm, respectively. The validation accuracies demonstrate that the MLP achieves a suitable result of 78.26%, whereas the results obtained for SVM and KNN are 74.4% and 76.6%, respectively [28]. Zuo et al. present a technique for the taxonomy of cardiac arrhythmia using a k-nearest neighbor classifier. The submitted method outperforms traditional KNN algorithms and produces more than 70% accuracy [29]. Besides that, an ANN-based architecture is applied to classify the Electrocardiography (ECG) records for cardiac arrhythmia taxonomy. It is claimed that the experimental results yield more than 87% classification accuracy [30]. Moreover, Persada et al. propose Best First and CsfSubsetEval for the feature selection process. The selected features are classified by using several classifiers, and the best precision is obtained by using the “RBF Classifier” in the combination of BFS and “CsfSubsetEval” techniques, producing 81% [31]. Jadhav et al. propose a modular neural network model for the binary classification (normal or abnormal) of arrhythmia dataset. The proposed model is claimed to attain 82.22% accuracy with the given dataset [32]. Further corresponding studies can be found in [33,34,35].

Moreover, a number of previous studies in the field of SPECTF classification are accessible. Srinivas et al. propose an SVM technique relying on sparsity-based dictionary learning. The proposed method presents 97.8% accuracy [36]. An alternative study offers a Bayesian network to select features. The method entails a vast number of features and produces 95.76% accuracy [37]. Cha et al. propose a new data description approach, namely support vector data description, which is assessed by employing datasets from the UCI repository. The method achieves almost 95% accuracy for the given dataset [38]. Furthermore, Liu et al. propose a new SVDD-based method. The proposed method offers 90% accuracy [39]. Previously, Cui et al. combined an improved version of k-nearest neighbors and the method is known as transductive confidence machine (TCM). The authors claim that this approach (TCM-IKNN) presents 90% accuracy with the UCI dataset [40]. Alternatively, a previous study on discretization approach, namely, “core-generating approximate minimum entropy discretization”, was also presented by [41]. This aims to control the lowermost entropy cuts in order to create discrete data points providing nonempty cores. The presented method is also confirmed by employing the UCI dataset and achieves 84% accuracy rate [41].

## 3. Material and Methods

The main contribution of this paper is to integrate a post processing procedure to a data classification framework. Accordingly, a strong deep learning framework combining sparse auto-encoders (SAEs) followed by a Softmax Classifier, a generalization of the binary form of the Logistic Regression method, is initially designed. The auto-encoder levels and the classifier level are stacked so as to be trained in a supervised approach based on a backpropagation algorithm. In order to increase the overall classification accuracy, a linear transformation function is integrated into the framework. This layer, in essence, improves the results obtained from DAEs based on a linear model. The critical issue here is to estimate the optimum parameter for the linear transformation model. A strong and reliable metaheuristic algorithm, PSO, is employed to approximate the most optimum model parameters. All these steps are detailed in the following sub sections.

### 3.1. Stacked Sparse Auto-Encoder

The stacked sparse auto-encoder (SSAE) is principally a neural network involving of a number of auto-encoders where each auto-encoder represents a layer and is trained in an unsupervised fashion using unlabeled data. The input of each auto-encoder is the output of the previous one. The training of an auto-encoder estimates the optimal parameters by using different algorithms which reduce the divergence between input x and output $\dot{x}$. The coding between input and output is represented by the equations illustrated below. Here, the input vector *x = (1, 2, 3, 4…, N),* is transformed into hidden representation “$\dot{x}$”, by employing a nonlinear model.

(1) $$\dot{x}=f\left(x\right)=M_{f}\left(W_{1}x+b_{1}\right)$$

(2) $$n_{1}^{\left(1\right)}=M_{f}\left(w_{11}^{\left(1\right)}x_{1}+\cdots w_{15}^{\left(1\right)}x_{5}+b_{1}^{\left(1\right)}\right)$$

(3) $$n_{i}^{\left(1\right)}=M_{f}\left(w_{i1}^{\left(1\right)}x_{1}+\cdots w_{i5}^{\left(1\right)}x_{5}+b_{i}^{\left(1\right)}\right)$$

Here $n_{i}^{\left(1\right)}$ refers to the ith neuron at the first layer for the architecture, M is an activation function, *w_i,_* and *b_i_* refer to weight matrix and the bias parameter, respectively.

The final mathematical model is illustrated in Equation (4):

(4) $$n_{w,b}\left(x\right)=M_{f}\left(w_{11}^{\left(2\right)}n_{1}^{\left(2\right)}+\cdots w_{15}^{2}n_{5}+\cdots +b_{1}^{\left(2\right)}\right)$$

**Figure A1:**
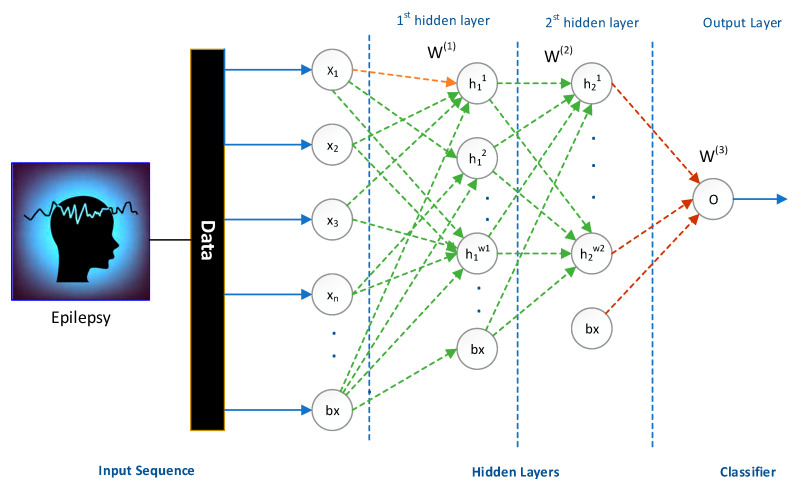
The model of stack a Stacked Sparse Auto-encoder (SSAE) with two hidden layers and a classifier (SoftMax).

The input x and output $\dot{x}$ discrepancy is represented by using a cost function. Several algorithms are used to find the optimum parameters of the network. The corresponding mathematical model can be seen in [25,42]. The model of Stacked Sparse Auto-encoder (SSAE), used in the proposed framework, is illustrated in Figure A1 and can be seen in Appendix A. The model has two hidden layers and a classifier layer (SoftMax). 

### 3.2. The Particle Swarm Optimization (PSO) Algorithm

PSO algorithms are considered as population-based metaheuristic algorithms proposed by [43,44,45,46]. These algorithms impersonate the social behavior of birds for problem solving.The PSO algorithm is set with a group of arbitrary solutions, representing the particles, and then it explores to approximate an optimal solution by updating the generations. In each iteration, every particle is modified by considering the two (best) values, namely local and global best values. The first best solution that is attained so far by the particle itself is denoted as the best local solution and is stored, known as “pbest” value. Then, the other, global, refers the best solution achieved thus far by a particle located in the population, and this best solution is a global best, known as “gbest” value. The particle updates the positions and velocity by employing Equations (5) and (6) after selecting the best two solutions.

(5) $$X_{k+1}^{i}=X_{k}^{i}+V_{k+1}^{i}$$

(6) $$V_{k+1}^{i}=wV_{k}^{i}+c_{1}r_{1}\left(P_{k}^{i}-X_{k}^{i}\right)+c_{2}r_{2}\left(P_{k}^{g}-X_{k}^{i}\right)$$

Here, $X_{k}^{i}$ represents particle position, $V_{k}^{i}$ represents particle velocity, $P_{k}^{i}$ represents the best “remembered” individual particle position (pbest), $P_{k}^{g}$ represents the best swarm position (gbest), $c_{1}$ and $c_{2}$. are cognitive and social parameters. Additionally, $r_{1},r_{2}$ are random parameters between (0,1) and w refers inertial coefficient (0,1). This manipulates convergence and “explore-exploit” trade-off in the PSO algorithm. PSO algorithms offers a number of advantages when compared with other optimization algorithms. PSO is a fast optimization algorithm and only needs few parameters for tuning. Especially, when PSO is compared with one of its main counterpart algorithms, Genetics Algorithm (GA), it should be noted that PSO can converge faster and needs fewer parameters to be configured.
**Algorithm 1.** Pseudo Code of PSO Algorithm.    For each particle        Set particles in a random manner    End    Do          Estimate the Local best “*pBest*” for each particle          If the “*pBest*” is enhanced      Update “*pBest*” value    End          Global Best (*gBest*) is updated as the best of “*pBests*”          For each particle           Estimate the velocity of particles via Equations (5) and (6)           Update the positions of the particles          End End

Accordingly, PSO is successfully applied in several fields, such as neural networks, optimization problems, etc. Algorithm 1 refers to the conventional PSO algorithm [47].

### 3.3. A New Deep Learning Framework Using Deep Auto-Encoders and a Linear Model Based on PSO

**Figure 1:**
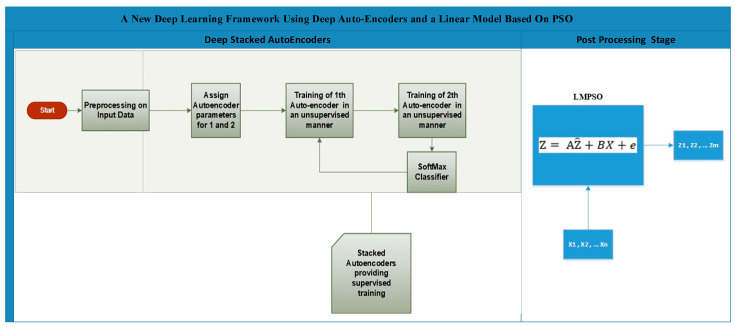
The Deep Learning Framework Based on a Linear Model and metaheuristic algorithm (PSO).

Suppose a trained deep stacked auto-encoder is used to classify an object into one of the “*M*” classes. The input layer of the deep stacked auto-encoder involves “*N*” neurons that are related to object features *X*_1_, *X*_2_, …, *X_N_*, and the output layer involves “*M*” neurons that stand for the expected output (class label) ${\hat{Z}}_{1}$, ${\hat{Z}}_{2}$, ${\hat{Z}}_{3}$, ${\hat{Z}}_{M}$ (see Figure 1). 

The deep auto-encoder involves two auto-encoders and Softmax, where the auto-encoders try to learn the high-level features from the input data X. The aim of using a number of auto-encoders is to reduce the number of features gradually. This is because dropping the number of features suddenly in one auto-encoder can lead to missing important features and affect the accuracy. The cost function of the stacked auto-encoders is represented as Equation (7).

(7) $$E=\frac{1}{N}\overset{N}{\underset{n=1}{\sum }}\overset{K}{\underset{k=1}{\sum }}{\left(x_{kn}-{\hat{x}}_{kn}\right)}^{2}+\lambda *\Omega_{weights}+\beta *\Omega_{sparsity}$$

Here, the error rate is denoted by E, the input features are illustrated by “*x*”, the reconstructed features are illustrated with “$\hat{x}$”, λ is the coefficient for the “L2 Weight Regularization”, *β* is the coefficient for “Sparsity Regularization”, and $\Omega_{weights}$ signifies the “L2 Weight Regularization”, which can be represented as shown in Equation (8).

(8) $$\Omega_{weights}=\frac{1}{2}\overset{L}{\underset{l}{\sum }}\overset{n}{\underset{j}{\sum }}\overset{k}{\underset{i}{\sum }}w_{ji}^{{\left(l\right)}^{2}}$$

Here, L presents the number of hidden layers, *n* is for the number of observations, and *k* indicates the variable number of the current training data. 

Finally, $\Omega_{sparsity}$ is the Sparsity Regularization parameter which adjusts the degree of sparsity of the output from the hidden layers, as illustrated in Equation (9).

(9) $$\Omega_{sparsity}=\overset{D\left(1\right)}{\underset{i=1}{\sum }}KL(\rho ||{\hat{\rho }}_{i})=\overset{D\left(1\right)}{\underset{i=1}{\sum }}\rho \;log(\rho ||{\hat{\rho }}_{i})+\left(1-\rho \right)log\left(\frac{1-\rho }{1-{\hat{\rho }}_{i}}\right)$$

Here, the desired value is represented by ρ, ${\hat{\rho }}_{i}$ symbolizes the average output activation of any neuron i, and *KL* represents the function, measuring the variation between two probability distributions based on the same data. Furthermore, the features that produce minimum cost in Equation (1) are selected and become input to Softmax, see Equation (10). Softmax is exploited as a classifier of the extracted features from X to the labels Z (see Figure 1).

(10) $$Q_{SoftMax\;}\left(z^{i}\right)=\frac{e^{z^{\left(i\right)}}}{\sum_{j=0}^{k}e^{z_{k}^{\left(i\right)}}}$$

Here the net input *z* is defined as

(11) $$z=\overset{m}{\underset{l=0}{\sum }}w_{l}\;x_{l}$$

Here, while *w* represents the weight vector, *x* symbolizes the feature vector of *lth* training sample. Essentially, the Softmax function calculates the probability of belonging to a class “j” for a training sample “*x^(i)^*” by taking into account the given weights and net input *z^(i)^*. Softmax is used without other classifiers because it is a transfer function and multiclass classifier which acts like an output layer to the previous auto-encoders. Then, the auto-encoders and Softmax layers are combined and trained by using a backpropagation algorithm in a supervised fashion to improve the performance of the network. 

Moreover, antithetically to previous deep learning applications, the output of the deep auto-encoder does not generate the final prediction but optimizes it by using a linear model [48]. Essentially, the performance of a deep networks is considered by the network’s structure, transfer function, and learning algorithm. Yet, a network classifier tends to be weak once it is designed based on an inappropriate structure. Essentially, there is no certain way to estimate a proper structure. A recent study proposed a linear model as a post processing layer based on Kalman Filter to improve overall classification performance [49]. Our study is inspired by this previous work and it employs the linear model so as to transform the predicted output of the network to a value close to the desired output via the linear combination of the object features and the expected output. This simple transformation can be considered as a post processing step, reducing the error of network and enhancing classification performance. A metaheuristic approach, PSO, is employed to optimize the parameters of the linear model. Overall, the parameters of the Linear model are calculated during the iteration of PSO algorithm. The linear model utilizes the predicted output of the deep network and the object features as input to estimate the class labels. The output of the DSAE $\hat{Z}$ is processed in a linear model by using *X*, coefficients *A, B* and the error rate e to produce the optimized result *Z* (see Equation (12)).

(12) $$Z=A\hat{Z}+BX+e$$

Here, A represents diagonal matrix *M × M* as shown in (13), *B* denotes *M × N* matrix as shown in (14), and e is for the error rate. Moreover, coefficients, namely *A* and *B*, are unknown for the linear model [50]. The values of A and B are estimated by using a PSO algorithm, and the parameters of PSO are selected depending on the problem type and input features.

(13) $$A=diag\;\begin{array}{ccc} {}[a_{11} & a_{22} & a_{MM}] \end{array}$$

(14) $$B=\left[\begin{array}{ccc} b_{11} & \cdots & b_{1N}\\ \vdots & \ddots & \vdots \\ b_{M1} & \cdots & b_{MN} \end{array}\right]$$

**Figure 2:**
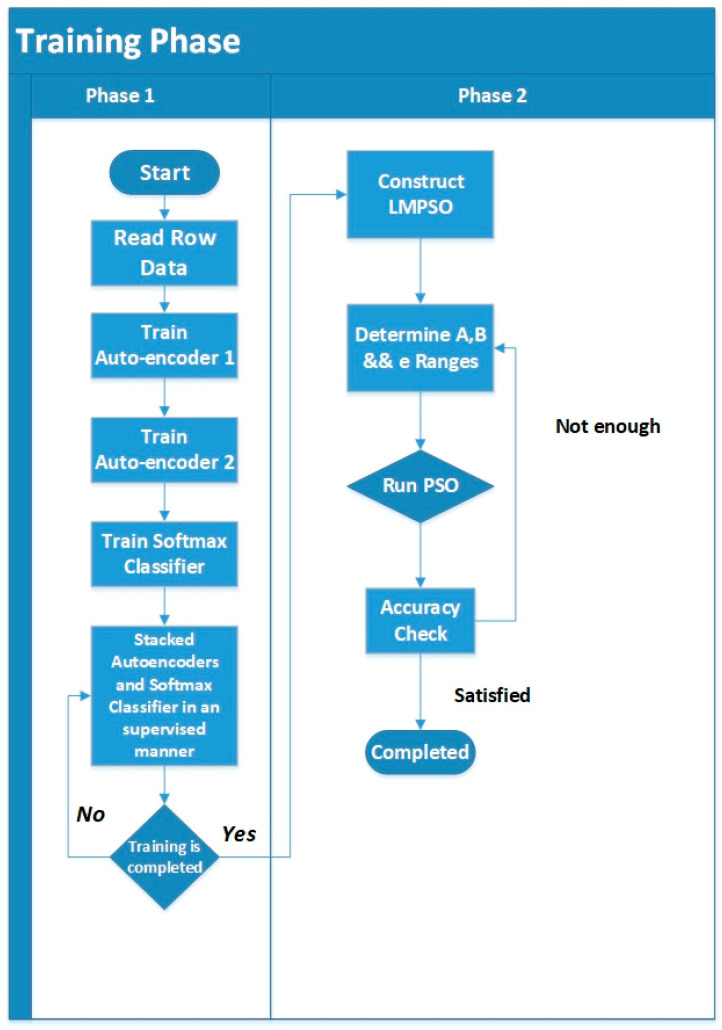
Training Flowchart for the Proposed Framework.

The details of the linear model mathematics are explained in [49], and the whole framework flowchart is illustrated in Figure 2.

In each iteration of PSO, the predicted Z is controlled by using MSE with optimal prediction *Q*, as illustrated in Equation (15).

(15) $$MSE=\frac{1}{m}\overset{m}{\underset{i=1}{\sum }}{\left(Q_{i}-Z_{i}\right)}^{2}$$

Here, m denotes the number of examples, *Q_i_* is the optimum class label for input features and MSE is the discrepancy rate between the $z_{i}$ and $Q_{i}$.

The MSE is represented as a cost function. PSO minimizes its value by estimating the best values for parameters *A*, *B* and *e.*

## 4. Experimental Results

The parameters calculated to improve the performance of the proposed framework are: “True Positive Rate” (Recall), “True Negative Rate” (TNR), “positive predictive value” (Precision), “negative predictive value” (NPV), “false positive rate” (FPR), “false discovery rate” (FDR), “miss rate” (MR), “accuracy” (ACC), “F1 score” (F1-s) and “Matthews correlation coefficient” (MCC). Definitions of these parameters can be seen authors’ previous work [25]. 

Each dataset has been divided into test and training sets according to the preliminary experiments and based on our previous studies. According to these, the Epileptic Seizure dataset is divided as 100 samples for training and the other 100 for testing, indicating 50% for test and 50% for training. The SPECTF Classification dataset, on the other hand, is arranged as 187 (70%) for training, and 87 (30%) for the testing process. The final dataset, the cardiac arrhythmias dataset, consists of 450 instances from 16 classes with 70% of those data employed for training and 30% for the testing procedures, respectively. Overfitting is a critical problem for classification models. In order to prevent overfitting, a random subsampling validation technique was applied during the training process. Following this, each experiment is repeated five times and the average of those experiments is registered. 

### 4.1. Epileptic Seizure

**Figure 3:**
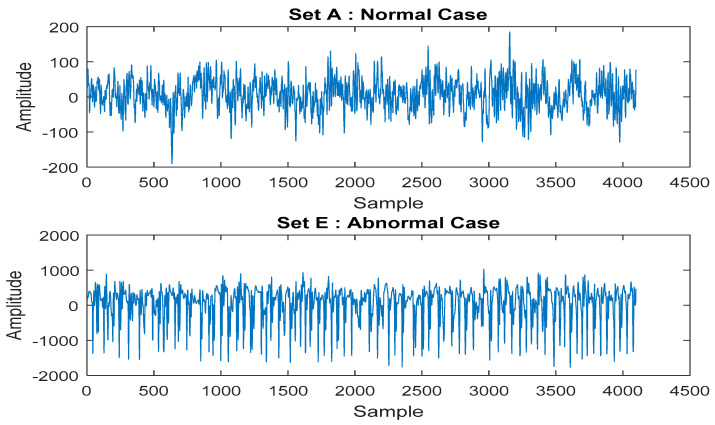
Datasets for Normal and Abnormal Cases.

The proposed framework is confirmed by employing a popular public dataset provided by Bonn University [51]. The dataset consists of 200 samples, with each sample consisting of 4096 features. The EEG data is split into two groups for training and testing procedures. Each group involves 100 examples, 50 of which are normal and the remaining 50 are abnormal. Those cases are illustrated in Figure 3. According to the framework, the first and second auto-encoders extract high-level features obtained from EEG signals and then diminish the number of features to 2007 and 112, respectively. Details of the parametric configuration of auto-encoders are shown in Table 1. Later, the Softmax layer classifies the extracted features as being normal and abnormal. 

**Figure 4:**
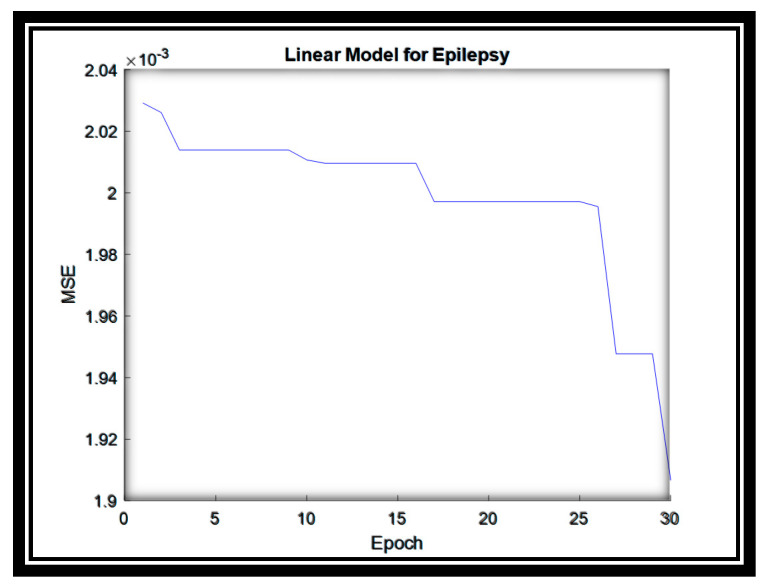
The MSE for the Linear System for Epilepsy dataset.

The linear model is then used to enhance the results, and the parameters of the linear model are estimated by using the PSO algorithm. The linear model parameters are estimated in 30 epochs and a reasonable MSE value is produced, as shown in Figure 4. Besides, the parameters of PSO are presented in Table 2. 

The test process is repeated five times with the same parameters and hidden layer values, but in each implementation the training and test data are arbitrarily designated to avoid overfitting. The average results of the dataset based on previously defined evaluation parameters is shown in Table 3. The corresponding table represents the results during the testing process. 

### 4.2. SPECTF Classification

The proposed framework is assessed by employing another benchmark dataset, namely, “SPECTF, (Single Proton Emission Computed Tomography) Heart datasets”, which is mainly presented in [52]. This dataset involves “normal” and “abnormal” classes that comprise more than 267 examples, with each of these instances consisting of 44 features. There exists 40 occurrences of each class at the training dataset, whereas the validation dataset contains “172 normal” and “15 abnormal” examples. As it is noted, auto-encoders can reduce the input dimension, and accordingly, the features in auto-encoders 1 and 2 are reduced step-by-step to 40 and 35, respectively, which essentially extracts high-level and sensitive features from input data.

The constraints of the auto-encoders are illustrated in Table 4. The parameters of the PSO algorithm are presented in Table 5.

The experimental results are evaluated by calculating the values of parameters, as presented in Table 6.

**Figure 5:**
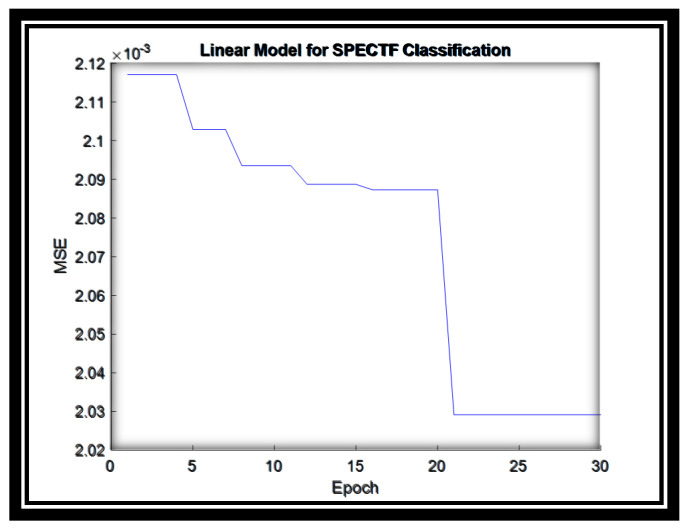
The MSE for the Linear System for SPECTF dataset.

For this dataset, the linear model parameters are converged in almost 20 epochs and produce “2.03” error value as illustrated in Figure 5.

### 4.3. Diagnosis of Cardiac Arrhythmia

The final benchmark dataset involves the data regarding cardiac arrhythmia, presented in [52]. This dataset consists of 450 instances from 16 different classes. Each class has 279 features. The proposed framework is trained for this dataset, according to which, the first Auto-Encoder is trained by employing an unsupervised approach and achieves a decrease in the number of features from 279 to 250. The output of the first one is passed to the second auto-encoder, which is also trained in an unsupervised manner. Afterwards, the number of features is reduced from 250 to 200. Essentially, those auto-encoders layers extract appropriate features in an unsupervised manner. The output is fed to Softmax Layer for multi class classification that helps to generate the classification probabilities. The whole architecture, on the other hand, propagates the error by using a backpropagation algorithm. This allows the framework to have supervised characteristics as aforementioned. Auto-encoder parameters for this dataset are shown in Table 7.

Table 8 presents the parameters of PSO which are employed to estimate the best parameters of the linear model. Table 9 demonstrates the proposed framework experimental performance regarding the performance evaluation parameters. 

**Figure 6:**
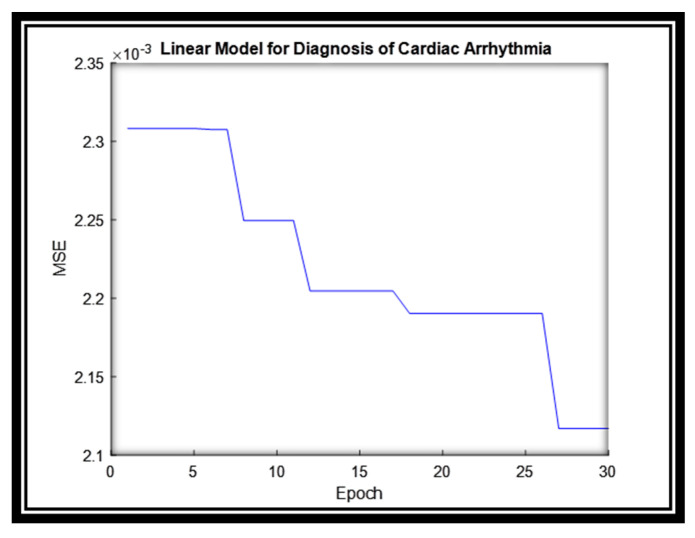
The MSE for the Linear System for Diagnosis of Cardiac Arrhythmia.

For this dataset, the linear model parameters are estimated in almost 28 epochs and produce 2.11 error rate as illustrated in Figure 6.

### 4.4. Statistical Significance Analysis of Algorithms in the Proposed Method

In applied machine learning, comparing the algorithms and proposing a final appropriate model for the presented problem is a common approach. Models are generally evaluated using resampling methods (k-fold cross-validation etc.). In these methods, mean performance scores are calculated and compared directly. This approach can give wrong ideas because it is difficult to understand whether the difference between mean performance scores is real or the result of a statistical chance. Statistical significance tests are proposed to overcome this problem and measure the likelihood of the samples with the assumption that they were selected from the equivalent distribution. If this assumption, or null hypothesis, is rejected (if a critical value is smaller than the significance level), it suggests that the difference in skill scores is statistically significant.

**Figure 7:**
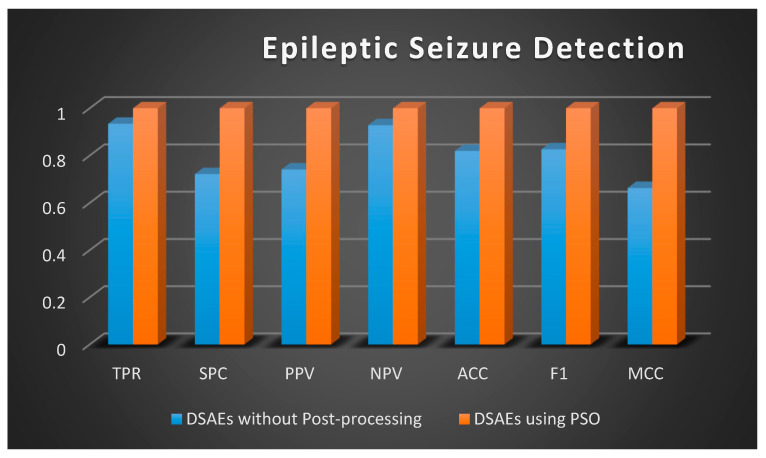
Graphical Representation of Performance Criteria for Epileptic Seizure Detection.

**Figure 8:**
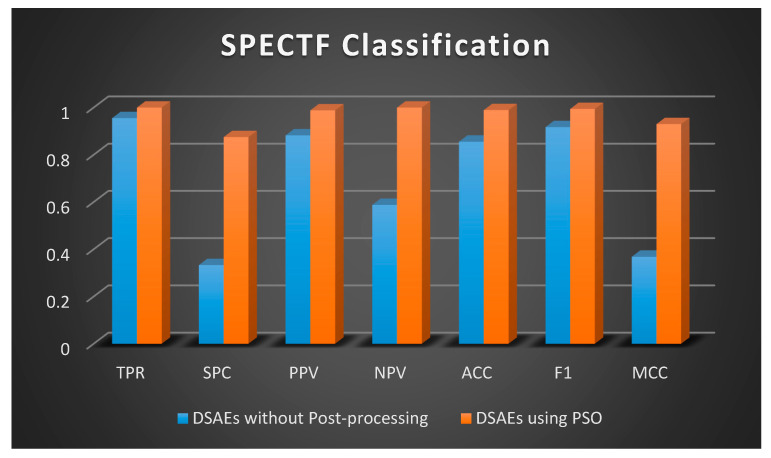
Graphical Representation of Performance Criteria for SPECTF Classification.

**Figure 9:**
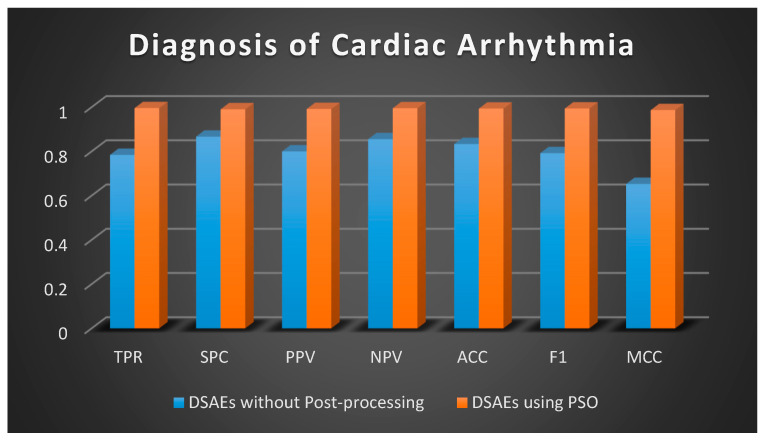
Graphical Representation of Performance Criteria for Diagnosis of Cardiac Arrhythmia.

Once the data is distributed normally, the two-sample *t*-test (regarding independent sets) and the paired *t*-test (for matched samples) are possibly considered the most extensively preferred methods in statistics for the assessment of differences between two samples [53]. A *t*-test is a type of statistical test that is employed to compare the means of two groups. A 2-tailed paired *t*-test is preferred in this study to compare the difference between the results without post-processing using PSO and the results after post-processing with PSO (Figure 7, Figure 8 and Figure 9) in order to evaluate if there is a statistically significant difference when the results are optimized. Two-tailed tests are able to identify differences in either path, greater or less than [54].

A 2-tailed paired *t*-test is applied in Excel on the two matched groups of epileptic seizure detection and *p*-value is calculated as 0.002463, that is, less than the standard level of significance (*p* < 0.05) so a statistically significant difference is noted on this data without using PSO and using PSO. The null hypothesis can be rejected since the sample data support the hypothesis that the population means are dissimilar.

A 2-tailed paired *t*-test is applied in Excel on the two matched groups of SPECTF classification and *p*-value is calculated as 0.020919, that is, less than the standard level of significance (*p* < 0.05) so a statistically significant difference is noted on this data without using PSO and using PSO. The null hypothesis can be rejected since the sample data support the hypothesis that the population means are dissimilar.

A 2-tailed paired *t*-test is applied in Excel on the two matched groups of diagnosis of cardiac arrhythmia and *p*-value is calculated as 0.000307, that is, less than the standard level of significance (*p* < 0.05) so a statistically significant difference is noted on this data without using PSO and using PSO. The null hypothesis can be rejected since the sample data support the hypothesis that the population means are dissimilar.

### 4.5. Performance Evaluation of the Framework Using Benchmark Datasets

The results of the proposed method, performed on benchmark datasets, are compared to several studies presented in this field. Then, the previous studies are analyzed to reveal the performance of the proposed framework. The comparison results for each dataset are detailed in Table 10, Table 11 and Table 12. Table 10 represents the comparison between the proposed framework and the leading state-of-the-art studies using Epileptic Seizure Dataset [51], whereas Table 11 involves the comparison based on SPECTF Dataset). Table 12, on the other hand, represents the performance comparison using Cardiac Arrhythmia Dataset. Details of both SPECTF and Cardiac Arrhythmia Datasets can be seen in [52].

#### 4.5.1. Epileptic Seizure Dataset 

According to the results shown in Table 10, the proposed framework presented better results than a number of studies [17,18,19,21,22,23,36] and presented the same results as other studies with a difference in the complexity and execution time. Peker et al. [24] propose traditional machine techniques which require a long processing time when compared with our proposed framework exactly in high-dimensional features such as epileptic seizure detection. Moreover, in a recent study, the authors propose to train DAEs using the Taguchi method for complex systems. According to this, the parameters are fitted manually when compared with our proposed framework that automatically optimizes the obtained results without needing to repeat experiments manually to obtain the best accuracy [25]. 

#### 4.5.2. SPECTF Dataset 

For this sub-section, results obtained from the proposed framework are compared with well-known studies in the field of SPECTF classification, as shown in Table 11. 

The proposed framework achieves better outcomes than all studies can be seen in [16,37,38,39,40,41,55].

#### 4.5.3. Cardiac Arrhythmia Dataset 

Finally, the proposed framework shows remarkable results when compared with well-known studies in the field of cardiac arrhythmia, as illustrated in Table 12.

Those studies can be seen in [28,29,30,31,32,33,34,35]. The results verify the advantage of the proposed system over previous relevant papers using the Cardiac Arrhythmia dataset. As previously mentioned, there exist 16 different classes for labelling the dataset. Accordingly, the proposed method accomplishes the best result when it is compared with the state-of-the-art studies.

## 5. Conclusions

This paper proposes a framework for data classification problems. This novel framework incorporates an efficient deep learning approach (DAE) and linear model trained by a metaheuristic algorithm (PSO). Despite their efficiency, DAEs may produce low performance when employed for complex problems, such as EEG signal classification and motion estimation. Accordingly, the overall goal of this framework is to increase the performance of the DAEs by integrating a post processing layer. This layer essentially optimizes the results obtained from DAEs based on a linear model trained by PSO algorithm. This metaheuristic approach is mainly employed to estimate the parameters of the linear model. As it has produced satisfactory results in various problems, it should be noted that it is easy to implement and involves quite a few parameters for tuning. 

Experimental results reveal that the proposed framework presents a number of advantages when compared with previous studies in the literature: learning using less data than other methods. The use of deep learning techniques leads to speeding up the processing time in high-dimensional features because it uses greedy layers as compared to convolutional techniques. The framework also proves that the overall performance of DAEs on complex problems can be enhanced by integrating a post processing layer. According to the results obtained, it is concluded that the introduced framework shows favorable results and can be adapted by researchers for any type of data classification problem. Additionally, as a future work, nonlinear and dynamic linear model systems can be proposed as a post-processing technique for enhancing the classification accuracy of the proposed framework. Moreover, additional optimization algorithms can be employed to train the models instead of PSO, such as the genetic algorithm, the gray-wolf optimization algorithm, the bat algorithm, and other classification models can be combined with linear and nonlinear models, such as support vector machines, naive Bayes or decision trees.

## Figures and Tables

**Table 1:** Auto-Encoder Parameters for Epileptic Seizure Detection.

Parameter	First Auto-Encoder	Second Auto-Encoder
Hidden Layer Size (HLS)	2007	112
Max Epoch Number (MEN)	420	110
L2 Regularization Parameter	0.004	0.002
Sparsity Regularization (SR)	4	2
Sparsity Proportion (SP)	0.14	0.12

**Table 2:** PSO Parameters for Epileptic Seizure Detection.

PSO Parameter	Value
Number of particles	50
Maximum iteration	30
Cognitive parameter	2
Social parameter	2
Min inertia weight	0.9
Max inertia weight	0.2

**Table 3:** Epileptic Seizure Detection Results.

Parameter	DSAEs without Post-Processing	DSAEs Using PSO
Recall	0.9348	1.0000
TNR	0.7222	1.0000
Precision	0.7414	1.0000
NPV	0.9286	1.0000
ACC	0.8200	1.0000
F1-s	0.8269	1.0000
MCC	0.6634	1.0000

**Table 4:** Auto-Encoder Parameters for Single Proton Emission Computed Tomography (SPECTF) Classification.

Parameter	Auto-Encoder 1	Auto-Encoder 2
Hidden Layer Size (HLS)	40	35
Max Epoch Number (MEN)	110	60
L2 Regularization Parameter	0.003	0.001
Sparsity Regularization (SR)	2	1
Sparsity Proportion (SP)	0.1	0.1

**Table 5:** PSO Parameters for SPECTF Classification.

PSO Parameter	Value
Number of particles	40
Maximum iteration	40
Cognitive parameter	2
Social parameter	2
Min inertia weight	0.9
Max inertia weight	0.2

**Table 6:** SPECTF Classification Results.

Parameter.	DSAEs without Post-Processing	DSAEs Using PSO
*Recall*	*0.9554*	*1.0000*
*TNR*	*0.3333*	*0.8750*
*Precision*	*0.8824*	*0.9884*
*NPV*	*0.5882*	*1.0000*
*ACC*	*0.8556*	*0.9893*
*F1-s*	*0.9174*	*0.9942*
*MCC*	*0.3686*	*0.9300*

**Table 7:** Auto-Encoder Parameters for Diagnosis of Cardiac Arrhythmia Using Post-Processing Technique.

Parameter	First Auto-Encoder	Second Auto-Encoder
Hidden Layer Size (HS)	250	200
Max Epoch Number (MEN)	130	109
L2 Weight Regularization	0.003	0.001
Sparsity Regularization (SR)	3	1
Sparsity Proportion (SP)	0.12	0.1

**Table 8:** PSO Parameters for Diagnosis of Cardiac Arrhythmia.

PSO Parameter	Value
Number of particles	60
Maximum iteration	45
Cognitive parameter	2
Social parameter	2
Min inertia weight	0.9
Max inertia weight	0.2

**Table 9:** Diagnosis of Cardiac Arrhythmia Results.

Parameter	DSAEs without Post-Processing	DSAEs Using PSO
Recall	0.7843	0.9959
TNR	0.8667	0.9904
Precision	0.8000	0.9918
NPV	0.8553	0.9952
ACC	0.8333	0.9934
F1-s	0.7921	0.9939
MCC	0.6531	0.9866

**Table 10:** Evaluation of the Proposed Framework with Leading State-of-the art Studies for Epileptic Seizure Detection.

Reference	Method	Accuracy
[36]	Time–frequency domain feature-RNN	99.6%
[17]	WT + ANN	92.0%
[18]	Discrete WT-mixture of expert model	94.5%
[19]	Entropy measures-ANFIS	92.22%
[20]	Time–frequency analysis—ANN	100%
[21]	Fast Fourier transform-DT	98.72%
[22]	WPD-PCA-GMM	99.00%
[23]	Entropies + HOS + Higuchi FD + Hurst exponent + FC	99.70%
[24]	DTCWT + CVANN-3	100%
[25]	Deep auto-encoder using Taguchi method	100%
[26]	Deep Auto-Encoder + Energy Spectral Density	100%
**Proposed Framework**	**Deep auto-encoder and linear model based PSO**	**100%**

**Table 11:** Comparison of SPECTF Classification Results.

Reference	Method	Accuracy
[38]	SVDD	82.7%
[39]	SVDD-based outlier detection	90%
[37]	K2	94.03%
	SDBNS	95.59%
	ECFBN	95.76%
[55]	mc-MKC	79.9%
	mc-SVM	79.1%
[40]	TCM-IKN N	90%
[41]	C-GAME + Johnson + c4.5	84.4%
	RMEP + Johnson + c4.5	81.7%
[16]	Sparsity-based dictionary learning + SVM	97.8%
[26]	Deep Auto-Encoder + Energy Spectral Density	96.79%
**Proposed Framework**	**Deep auto-encoder and linear model based PSO**	**98.93%**

**Table 12:** Comparison the performance of the framework on Cardiac Arrhythmia Dataset.

Reference	Method		Accuracy
	Feature Extraction Technique	Classifier	
[27]	Enhanced F-score and sequential forward search	k-NN SVM	74% 69%
[28]	Wrapper method	MLP k-NN SVM	78.26% 76.6% 74.4%
[29]	PCA	Kernel difference weighted k-NN	70.66%
[30]	-	MLP+ Static backpropagation algorithm	86.67%
[31]	Best First and CsfSubsetEval	RBF	81%
[32]	-	Modular neural network model	82.22%
[33]	-	ANN models + Static backpropagation algorithm + momentum learning rule	86.67%
[34]	One-against-all	SVM	73.40%
[35]	-	Resampling strategy based random forest (RF) ensemble classifier	90%
[26]	Energy Spectral Density + Deep Auto-Encoders	Softmax	99.1%
Proposed Framework	Deep auto-encoder and linear model based PSO	Softmax	99.27%

## References

[1] Xu M., Fralick D., Zheng J.Z., Wang B., Tu X.M., Feng C. (2017). The Differences and Similarities between Two-Sample *t*-test and Paired *t*-test. Shanghai Arch. Psychiatry.

[2] Sze V., Chen Y.-H., Yang T.-J., Emer J.S. (2017). Efficient Processing of Deep Neural Networks: A Tutorial and Survey. Proc. IEEE.

[3] Luckow A., Cook M., Ashcraft N., Weill E., Djerekarov E., Vorster B. Deep learning in the automotive industry: Applications and tools. Proceedings of the 2016 IEEE International Conference on Big Data (Big Data).

[4] Memisevic R. Deep learning: Architectures, algorithms, applications. Proceedings of the 2015 IEEE Hot Chips 27 Symposium (HCS).

[5] Chu L.W. (2012). Alzheimer’s disease: Early diagnosis and treatment. Hong Kong Med. J..

[6] Pushkar B., Paul M. Early Diagnosis of Alzheimer’s Disease: A Multi—Class Deep Learning Framework with Modified k- sparse Autoencoder Classification. Proceedings of the 2016 International Conference on Image and Vision Computing New Zealand (IVCNZ).

[7] Tong H., Liu B., Wang S. (2018). Software defect prediction using stacked denoising autoencoders and two-stage ensemble learning. Inf. Softw. Technol..

[8] Kuo J.Y., Pan C.W., Lei B. Using stacked denoising autoencoder for the student droupout predication. Proceedings of the 2017 IEEE International Symposium on Multimedia (ISM).

[9] Xiong Y., Zuo R. (2016). Recognition of geochemical anomalies using a deep autoencoder network. Comput. Geosci..

[10] Salaken S.M., Khosravi A., Khatami A., Nahavandi S., Hosen M.A. Lung cancer classification using deep learned features on low population dataset. Proceedings of the 2017 IEEE 30th Canadian Conference on Electrical and Computer Engineering (CCECE).

[11] Khatab Z.E., Hajihoseini A., Ghorashi S.A. (2017). A Fingerprint Method for Indoor Localization Using Autoencoder Based Deep Extreme Learning Machine. IEEE Sens. Lett..

[12] Khan U.M., Kabir Z., Hassan S.A., Ahmed S.H. A Deep Learning Framework Using Passive Wi-Fi Sensing for Respiration Monitoring. Proceedings of the GLOBECOM 2017—2017 IEEE Global Communications Conference.

[13] Tang X.-S., Hao K., Wei H., Ding Y. (2017). Using line segments to train multi-stream stacked autoencoders for image classification. Pattern Recognit. Lett..

[14] Yin C., Zhu Y., Fei J., He X. (2017). A Deep Learning Approach for Intrusion Detection Using Recurrent Neural Networks. IEEE Access.

[15] Yu Z., Tan E.-L., Ni D., Qin J., Chen S., Li S., Lei B., Wang T. (2017). A Deep Convolutional Neural Network-Based Framework for Automatic Fetal Facial Standard Plane Recognition. IEEE J. Biomed. Health Inform..

[16] Srinivas M., Bharath R., Rajalakshmi P., Mohan C.K. Multi-level classification: A generic classification method for medical datasets. Proceedings of the 2015 17th International Conference on E-health Networking, Application & Services (HealthCom).

[17] Subasi A., Erçelebi E. (2005). Classification of EEG signals using neural network and logistic regression. Comput. Methods Programs Biomed..

[18] Subasi A. (2007). EEG signal classification using wavelet feature extraction and a mixture of expert model. Expert Syst. Appl..

[19] Kannathal N., Choo M.L., Acharya U.R., Sadasivan P. (2005). Entropies for detection of epilepsy in EEG. Comput. Methods Programs Biomed..

[20] Tzallas A.T., Tsipouras M.G., Fotiadis D.I. (2007). Automatic Seizure Detection Based on Time-Frequency Analysis and Artificial Neural Networks. Comput. Intell. Neurosci..

[21] Polat K., Güneş S. (2007). Classification of epileptiform EEG using a hybrid system based on decision tree classifier and fast Fourier transform. Appl. Math. Comput..

[22] Acharya U.R., Sree S.V., Alvin A.P.C., Suri J.S. (2012). Use of principal component analysis for automatic classification of epileptic EEG activities in wavelet framework. Expert Syst. Appl..

[23] Acharya U.R., Sree S.V., Ang P.C.A., Yanti R., Suri J.S. (2012). Application of non-linear and wavelet based features for the automated identification of epileptic EEG signals. Int. J. Neural Syst..

[24] Peker M., Şen B., Delen D. (2015). A Novel Method for Automated Diagnosis of Epilepsy Using Complex-Valued Classifiers. IEEE J. Biomed. Heal. Inform..

[25] Karim A.M., Güzel M.S., Tolun M.R., Kaya H., Çelebi F.V. (2018). A New Generalized Deep Learning Framework Combining Sparse Autoencoder and Taguchi Method for Novel Data Classification and Processing. Math. Probl. Eng..

[26] Karim A.M., Serdar G.M., Tolun M.R., Kaya H., Çelebi F.V. (2019). A new framework using deep auto-encoder and energy spectral density for medical waveform data classification and processing. Biocybern. Biomed. Eng..

[27] Niazi K.A.K., Khan S.A., Shaukat A., Akhtar M. Identifying best feature subset for cardiac arrhythmia classification. Proceedings of the 2015 Science and Information Conference (SAI).

[28] Mustaqeem A., Anwar S.M., Majid M., Khan A.R. Wrapper method for feature selection to classify cardiac arrhythmia. Proceedings of the 39th Annual International Conference of the IEEE Engineering in Medicine and Biology Society (EMBC).

[29] Zuo W., Lu W., Wang K., Zhang H. (2008). Diagnosis of cardiac arrhythmia using kernel difference weighted KNN classifier. Comput. Cardiol..

[30] Jadhav S.M., Nalbalwar S.L., Ghatol A.A. ECG arrhythmia classification using modular neural network model. Proceedings of the IEEE EMBS Conference on Biomedical Engineering and Sciences (IECBES).

[31] Persada A.G., Setiawan N.A., Nugroho H. Comparative study of attribute reduction on arrhythmia classification dataset. Proceedings of the International Conference on Information Technology and Electrical Engineering (ICITEE).

[32] Jadhav S.M., Nalbalwar S.L., Ghatol A. Artificial Neural Network based cardiac arrhythmia classification using ECG signal data. Proceedings of the International Conference on Electronics and Information Engineering.

[33] Jadhav S.M., Nalbalwar S.L., Ghatol A.A. Artificial Neural Network Based Cardiac Arrhythmia Disease Diagnosis. Proceedings of the International Conference on Process. Automation, Control. and Computing.

[34] Kohli N., Verma N.K., Roy A. SVM based methods for arrhythmia classification in ECG. Proceedings of the International Conference on Computer and Communication Technology (ICCCT).

[35] Özçift A. (2011). Random forests ensemble classifier trained with data resampling strategy to improve cardiac arrhythmia diagnosis. Comput. Biol. Med..

[36] Srinivasan V., Eswaran C., Sriraam A.N. (2005). Artificial Neural Network Based Epileptic Detection Using Time-Domain and Frequency-Domain Features. J. Med. Syst..

[37] Wei J., Yu H., Wang J. The research of Bayesian method from small sample of high-dimensional dataset in poison identification. Proceedings of the IEEE 4th International Conference on Software Engineering and Service Science.

[38] Cha M., Kim J.S., Baek J.-G. (2014). Density weighted support vector data description. Expert Syst. Appl..

[39] Liu B., Xiao Y., Cao L., Hao Z., Deng F. (2013). SVDD-based outlier detection on uncertain data. Knowl. Inf. Syst..

[40] Cui L.-l., Zhu H.-c., Zhang L.-k., Luan R.-p. (2010). Improved kNearest Neighbors Transductive Confidence Machine for Pattern Recognition. IEEE Int. Conf. Comput. Des. Appl..

[41] Tian D., Zeng X.-J., Keane J. (2011). Core-generating approximate minimum entropy discretization for rough set feature selection in pattern classification. Int. J. Approx. Reason..

[42] Zeng N., Zhang H., Song B., Liu W., Li Y., Dobaie A.M. (2018). Facial expression recognition via learning deep sparse autoencoders. Neurocomputing.

[43] Kennedy J., Eberhart R. Particle swarm optimization. Proceedings of the IEEE International Conference on Neural Networks (ICNN’95).

[44] Harman R. (1995). A Very Brief Introduction to Particle Swarm Optimization.

[45] Kaveh A., Nasrollahi A. (2014). A new probabilistic particle swarm optimization algorithm for size optimization of spatial truss structures. Int. J. Civ. Eng..

[46] Ding W., Lin C.-T., Cao Z. (2018). Deep Neuro-Cognitive Co-Evolution for Fuzzy Attribute Reduction by Quantum Leaping PSO With Nearest-Neighbor Memeplexes. IEEE Trans. Cybern..

[47] Serdar G.M., Kara M., Beyazkılıç M.S. (2017). An adaptive framework for mobile robot navigation. Adapt. Behav..

[48] Rizvi S.Z., Abbasi F., Velni J.M. Model Reduction in Linear Parameter-Varying Models using Autoencoder Neural Networks. Proceedings of the Annual American Control Conference (ACC).

[49] Siswantoro J., Prabuwono A.S., Abdullah A., Idrus B. (2016). A linear model based on Kalman filter for improving neural network classification performance. Expert Syst. Appl..

[50] Noy D., Menezes R. (2018). Parameter estimation of the Linear Phase Correction model by hierarchical linear models. J. Math. Psychol..

[51] Andrzejak R.G., Lehnertz K., Mormann F., Rieke C., David P., Elger C.E. (2001). Indications of nonlinear deterministic and finite-dimensional structures in time series of brain electrical activity: Dependence on recording region and brain state. Phys. Rev. E.

[52] Dua D., Karra T. (2017). Machine Learning Repository.

[53] Xu G., Fang W. Shape retrieval using deep autoencoder learning representation. Proceedings of the 13th International Computer Conference on Wavelet Active Media Technology and Information Processing (ICCWAMTIP).

[54] Kim T.K. (2015). T test as a parametric statistic. Korean J. Anesthesiol..

[55] Kumar R., Chen T., Hardt M., Beymer D., Brannon K., Syeda-Mahmood T. Multiple Kernel Completion and its application to cardiac disease discrimination. Proceedings of the IEEE 10th International Symposium on Biomedical Imaging.

